# Diuretic Use Among Hemodialysis Patients in Lebanon: A Multicenter Observational Retrospective Study

**DOI:** 10.1155/ijne/4348593

**Published:** 2025-12-17

**Authors:** Iqbal Fahs, Faten Ezzeddine, Mariam Dabbous, Jihan Safwan, Fouad Sakr, Mohamad Rahal

**Affiliations:** ^1^ School of Pharmacy, Lebanese International University, Beirut, Lebanon, liu.edu.lb; ^2^ RITMES-E4 Research Unit (Epidemiological, Microbiological, Molecular, and Phylogenomic Surveillance of Infectious Diseases), Institute of Research for Development (IRD), University Hospital Institute Méditerranée-Infection, Aix-Marseille University, Marseille, France, univ-amu.fr; ^3^ INSPECT-LB INSPECT-LB (National Institute of Public Health, Clinical Epidemiology, and Toxicology-Lebanon), Beirut, Lebanon; ^4^ EpiMaCT-Epidemiology of Chronic Diseases in Tropical Zone, Inserm U1094, IRD UMR270, CHU Limoges, Institute of Epidemiology and Global Health-Michel Dumas, OmegaHealth, University of Limoges, Limoges, France, unilim.fr; ^5^ Aging Research Team, Center for Epidemiology and Research in POPulation Health (CERPOP), Inserm, University of Toulouse (Formerly UPS–Toulouse III), Toulouse, 31000, France, inserm.fr; ^6^ University of Toulouse (Formerly UPS–Toulouse III), Toulouse, 31062, France

**Keywords:** clinical outcomes, diuretic use, hemodialysis patients, Lebanon, loop diuretics, optimal dose

## Abstract

**Background:**

Despite their fundamental role in managing volume overload, diuretic use in hemodialysis patients is inconsistent. This study aims to examine diuretic use, compare clinical outcomes between diuretic users and nonusers, and identify predictive factors among hemodialysis patients in Lebanon.

**Method:**

This was a multicenter retrospective observational study. Patients’ data were retrieved from eight large dialysis centers in Lebanon. Descriptive and bivariate analyses were performed, followed by multivariable logistic regression to examine the association of diuretic use with the sociodemographic, hemodialysis parameters, and clinical outcomes of patients.

**Results:**

A total of 250 patients were included in the study. Diuretics were utilized among 38.4% of the patients, all of whom were on loop diuretics, primarily furosemide (71.3%). The mean furosemide equivalent dose was 153.56 ± 122.57 mg per day (range 20–500 mg). Only 28.7% and 16.1% were on furosemide equivalent doses of at least 250 mg and 320 mg per day, respectively. Diuretic users were more likely to have a residual kidney function (ORa = 23.189, 95% CI: 5.129–104.831, *p* < 0.001), a shorter dialysis vintage (ORa = 0.892, 95% CI: 0.472–0.958, *p* = 0.046), and a higher postdialysis systolic blood pressure (ORa = 13.760, 95% CI: 10.381–18.232, *p* = 0.026) compared to nonusers. Other hemodialysis parameters and clinical outcomes did not differ between diuretic users and nonusers. Furosemide equivalent doses of at least 250 mg per day were significantly associated with lower predialysis systolic blood pressure and dry weight, while doses of 320 mg and more per day were significantly associated with fewer intradialytic hypotension episodes, lower predialysis systolic blood pressure, dry weight, and interdialytic weight gain (all *p* < 0.05).

**Conclusion:**

This study reveals suboptimal dosing despite a relatively high prevalence of utilization of diuretics among hemodialysis patients. Most hemodialysis parameters and clinical outcomes do not differ between diuretic users and nonusers. Higher diuretic doses are associated with improved clinical outcomes, emphasizing the need for further research to optimize dosing practices in this population.

## 1. Introduction

End‐stage renal disease (ESRD) is characterized by irreversible loss of renal function necessitating renal replacement therapy for survival [1]. The prevalence of ESRD is on the rise worldwide, driven by aging populations and increasing prevalence of hypertension and diabetes mellitus [2, 3]. ESRD imposes a significant healthcare burden, with an estimated global prevalence of nearly 4 million individuals undergoing renal replacement therapy [4]. Among the various modalities available for renal replacement therapy, hemodialysis stands as a cornerstone, providing vital support in managing ESRD patients [5]. It accounts for approximately 69% of all renal replacement therapy and 89% of all dialysis [6].

Despite its life‐sustaining benefits, hemodialysis is not devoid of complications. It can negatively affect the general health and well‐being of the patients, physical performance, and mental status [7]. One of the common acute complications is intradialytic hypotension that occurs in almost one‐third of hemodialysis sessions [8]. Caused primarily by aggressive ultrafiltration in response to interdialytic weight gain, intradialytic hypotension can lead to myocardial stunning and cardiac arrhythmias and is associated with increased risk for death [9, 10]. On the other hand, intradialytic hypertension has been identified in up to 15% of hemodialysis patients and is associated with higher hospitalization rates and decreased survival [11]. Moreover, the intermittent nature of the hemodialysis regimen results in fluid overload, which is associated with hypertension and cardiac dysfunction. Consequently, it is a major risk factor for both all‐cause and cardiovascular mortality [12]. Dialysis corrects electrolyte imbalances; however, it can also precipitate them [13]. Among noncompliant hemodialysis patients, the most predominant electrolyte abnormality is hyperkalemia, alongside hyperphosphatemia, hypermagnesemia, hyponatremia, and hypocalcemia [10, 13]. Therefore, effective management of these complications is crucial for optimizing patient outcomes and improving quality of life [14].

While being widely prescribed to manage fluid overload and hypertension in patients with chronic kidney disease [15], diuretic use is far less common in dialysis patients [16, 17]. However, achieving euvolemia is important in dialysis patients [18]. Utilizing oral diuretics in hemodialysis patients with residual kidney function is beneficial to improve interdialytic fluid status [18]. They can reduce interdialytic weight gain with a consequent reduction in ultrafiltration rates, leading to fewer episodes of intradialytic hypotension [19]. Furthermore, loop diuretics can control hyperkalemia risk in hemodialysis patients [16]. It was revealed by two observational studies that diuretic users have lower rates of volume‐related morbidity and mortality in hemodialysis‐dependent kidney failure [16, 20]. Despite their established benefits, diuretics are often discontinued upon dialysis initiation [18]. Definitive conclusions about maintaining diuretic therapy in dialysis patients are hindered by confounding effects related to residual renal function. [18, 21]. Other factors include interpatient variability in response to therapy, absence of consensus on dosing strategies, and potential adverse effects [18, 22].

Lebanon is a developing country in the Middle East region. ESRD poses a substantial public health issue in Lebanon, with a growing prevalence of individuals requiring dialysis estimated at 777 patients per million population (pmp) against 410 dialysis patients pmp worldwide [23, 24]. Despite the increasing demand for hemodialysis services, there is a scarcity of data regarding pharmacotherapy of hemodialysis patients. Without robust information, healthcare providers may struggle to make informed decisions for this patient population. The objectives of this study were to examine the utilization of diuretics among Lebanese patients with ESRD, compare hemodialysis parameters and clinical outcomes among diuretic users and nonusers, and identify predicting factors of diuretic utilization. To our knowledge, this is the first multicenter study from Lebanon describing diuretic prescribing patterns and dose‐stratified clinical outcomes among hemodialysis patients, complementing prior work from high‐income settings [21]. The aim is to enhance understanding of the utilization of diuretics among hemodialysis patients in Lebanon, ultimately informing national and, if possible, regional clinical practice and improving patient care.

## 2. Methods

### 2.1. Study Design and Participants

A multicenter retrospective observational study was conducted between January and October 2023 at 8 large dialysis centers distributed all over the Lebanese districts (2 from Beirut, 2 from Mount Lebanon, 1 from Bekaa, 2 from South Lebanon, and 1 from North Lebanon). A cover letter that explained the study objectives along with the research proposal was shared randomly with 30 hospitals that have dialysis centers, out of which only 8 agreed to participate. Medical records were reviewed to retrieve patients’ data for the previous 6 months. All adult patients aged at least 18 years of age who were on hemodialysis for the last 6 months were eligible for inclusion. On the other hand, transplant patients and those with cognitive abnormalities or on complete bed rest were excluded.

### 2.2. Ethical Approval

This study was conducted in accordance with the ethical standards of the Declaration of Helsinki (2013 revision). Ethical approval was obtained from the Ethics and Research Committee of the School of Pharmacy at the Lebanese International University (Approval No. 2023ERC‐130‐LIUSOP, January 2, 2023) and from the institutional review boards of all participating hospitals. Written informed consent was obtained from all participants prior to inclusion. Patient confidentiality and data privacy were strictly maintained throughout the study.

### 2.3. Study Measures and Outcomes

The questionnaire was developed based on previous research studies [16, 20, 21, 25]. The questionnaire was reviewed by all authors to ensure content validity and relevance. Data were primarily collected from patients’ medical records, and additional information not documented in the files was obtained directly from patient interviews conducted by the study investigators. A pilot study prior to data collection was performed on 10 cases to determine the questionnaire’s consistency, and their results were not included in the analysis of this study. Accordingly, minor adjustments were made, resulting in the final version of the survey comprising two main sections.

The first part of the data collection sheet retrieved the sociodemographic factors, including age, gender, marital status, educational level, residence, occupation, household crowding index (defined as the total number of co‐residents per household, excluding newborns, divided by the total number of rooms, excluding kitchen and bathrooms), monthly income, smoking status, alcohol status, family history, comorbidities, and ERSD etiology. The second part gathered information regarding dialysis vintage, number of dialysis sessions per week, vascular access, diuretic use, type of diuretic, dose of the diuretic, furosemide equivalent dose, and clinical parameters such as potassium level, total number of hospitalizations, total number of intradialytic hypotension episodes, urine output, pre‐ and postdialysis blood pressure, ultrafiltration volume, dry weight, pre‐ and postdialysis weight, and interdialytic weight gain in each session for the past 6 months.

The furosemide equivalent dose was computed based on the established equivalency of 40 mg of oral furosemide to 1 mg of oral bumetanide [26]. The average of potassium level, pre‐ and postdialysis blood pressure, ultrafiltration volume, dry weight, and pre‐ and postdialysis weight were calculated using values retrieved from medical files for the past 6 months. An intradialytic hypotension episode was defined as nadir systolic blood pressure < 90 mmHg for patients with a predialysis value of < 160 mmHg or nadir systolic blood pressure < 100 mmHg for patients with a predialysis value of ≥ 160 mmHg [19]. Dry weight was defined as the lowest weight a patient can tolerate without the development of symptoms or hypotension [27]. Average interdialytic weight gain was computed as the difference between the average postdialysis weight of one session and the predialysis weight of the next dialysis session [16].

### 2.4. Sample Size Calculation

The CDC Epi Info software was used to calculate the minimal sample size. The population size was set at 5,353,930. According to the registration database of the Ministry of Public Health in Lebanon, the prevalence of dialysis cases is estimated at 777 patients per million population (0.00777 per 100 individuals) [23, 28]. Based on these calculations, the expected frequency was set at 1%. Accordingly, a minimum sample of 15 hemodialysis patients was required to allow for statistical analysis and produce a 95% confidence interval (CI) with a 5% margin of error. However, to allow for additional multivariable analysis, it was decided to increase the sample size and include all patients on hemodialysis at the dialysis centers where the study was conducted.

### 2.5. Data Analysis

Data were analyzed using the IBM Statistical Package for Social Sciences program Version 25 (IBM‐SPSS Statistics 25). A descriptive analysis was performed using absolute frequencies and percentages for categorical variables and means with standard deviations for quantitative variables. Bivariate analysis was conducted to compare sociodemographic characteristics, hemodialysis parameters, and clinical outcomes between diuretic users and nonusers. Bivariate analysis utilized the independent sample *T*‐test for the comparison of two groups and one‐way analysis of variances (ANOVAs) for the comparison among multiple groups. The Levene’s test was applied to assess variance homogeneity, resorting to the corrected *T*‐test when variances were not homogeneous. Additionally, the chi‐square was used to compare categorical variables. Afterward, a multivariable logistic regression analysis was performed, taking the diuretics use as the dependent variable and variables with *p* < 0.2 in the bivariate analysis as independent variables. Results were reported as adjusted odds ratio (ORa).

Thereafter, a subgroup analysis was conducted to evaluate the differential effects and clinical outcomes of diuretics across specific dosage thresholds, with furosemide doses set at 80 mg, 250 mg, and 320 mg per day. These cutoff points were chosen based on the clinical condition of the patients, as doses lower than 80 mg per day are ineffective for those with advanced chronic kidney disease [29]. Furthermore, previous research has shown that daily doses of at least 250 mg of furosemide can increase urine volume by 60% in chronic hemodialysis patients [30]. Additionally, in cases of severe renal insufficiency, it was demonstrated that a daily dose of 320–400 mg is required for maximal natriuresis [26]. By stratifying patients into these subgroups, the analysis aimed to determine whether higher daily diuretic doses confer additional benefits to hemodialysis patients. Bivariate analysis using an independent sample *T*‐test was performed to compare the means of clinical outcomes across the specified dosage thresholds. The level of significance was set at *p* < 0.05 with a predetermined margin of error of 5%.

## 3. Results

### 3.1. Sociodemographic Characteristics

The mean age of the 250 studied participants was 59.65 ± 13.86 years. The gender distribution was nearly equal, with 52% male and 48% female. A majority of patients were married (67.6%), and most resided in South Lebanon (34.8%) and Beirut (27.2%). Educational attainment was low, with 68.4% having primary education or less. Financially, 44.8% of households earned less than $300 monthly, and a significant number of the patients (85.2%) were not working. Regarding health behaviors, 35.2% were current smokers, and 84% consumed caffeine. Only 10.4% consumed alcohol. In terms of comorbidities, 74% had hypertension, 30% had diabetes mellitus, and 27.6% had heart disease. The leading causes of ESRD were hypertension (56%) and a combination of hypertension and diabetes mellitus (15.2%). Table 1 demonstrates the sociodemographic baseline characteristics of the studied population.

**Table 1:** Sociodemographic baseline characteristics of the participants.

Variable	M ± SD or *N* (%) (*N* = 250)
Age (years)	59.65 ± 13.86
Gender	
Male	130 (52)
Female	120 (48)
Marital status	
Single	27 (10.8)
Married	169 (67.6)
Divorced/widowed	54 (21.6)
Residence	
Beirut	68 (27.2)
Mount Lebanon	32 (12.8)
Bekaa	24 (9.6)
South Lebanon	87 (34.8)
North Lebanon	39 (15.6)
Education level	
Primary or less	171 (68.4)
High school	47 (18.8)
University/postgraduate studies	32 (12.8)
Household crowding index	1.17 ± 0.76
Household income	
Less than 300$	112 (44.8)
300$–500$	100 (40)
More than 500$	38 (15.2)
Occupation	
Nonworking	213 (85.2)
Working	37 (14.8)
Smoking status	
Nonsmoker	110 (44.0)
Ex‐smoker	52 (20.8)
Current smoker	88 (35.2)
Caffeine status	
Yes	210 (84)
No	40 (16)
Alcohol status	
Yes	26 (10.4)
No	224 (89.6)
Potassium‐restricted diet	
Yes	87 (34.8)
Most of the time	103 (41.2)
No	60 (24)
Family history	
Chronic kidney disease	69 (27.6)
Hypertension	105 (42)
Diabetes mellitus	88 (35.2)
Comorbidities	
Hypertension	185 (74)
Diabetes mellitus	75 (30)
Heart disease	69 (27.6)
ESRD etiology	
HTN	140 (56)
DM	11 (4.4)
HTN and DM	38 (15.2)
Others	61 (24.4)

### 3.2. Hemodialysis Characteristics and Clinical Parameters

Table 2 reveals the hemodialysis characteristics and clinical parameters of the participants. The average duration of dialysis treatment was 5.53 years. Most patients had dialysis sessions three times per week (58.8%). Urine output was present in 68.4% of patients, and 85.2% used an arteriovenous fistula for vascular access. The average number of hospitalizations during the past 6 months was 0.17 per patient, while intradialytic hypotension episodes averaged 2.93 per patient. Key clinical parameters included an average potassium level of 5.41 mEq/L, predialysis systolic blood pressure of 138.1 mmHg, postdialysis systolic blood pressure of 118.9 mmHg, dry weight of 69.12 kg, interdialytic weight gain of 2.41 kg, and an ultrafiltration volume of 2.76 L per session.

**Table 2:** Hemodialysis characteristics and clinical parameters of the participants.

Variable	M ± SD or *N* (%) (*N* = 250)
Dialysis vintage (years)	5.53 ± 4.9
Number of dialysis sessions per week	
2x per week	103 (41.2)
3x per week	147 (58.8)
Urine output	
Yes	171 (68.4)
No	79 (31.6)
Vascular access	
Arteriovenous fistula	213 (85.2)
Venous catheter access	37 (14.8)
Total number of hospitalizations per patient per 6 months	0.17 ± 0.49
Total number of intradialytic hypotension episodes per patient per 6 months	2.93 ± 2.98
Average potassium levels (mEq/L)	5.41 ± 0.73
Average predialysis systolic blood pressure (mmHg)	138.12 ± 17.60
Average postdialysis systolic blood pressure (mmHg)	118.91 ± 13.17
Average dry weight (kg)	69.12 ± 15.91
Average interdialytic weight gain (kg)	2.41 ± 1.00
Average ultrafiltration volume (L)	2.76 ± 1.00

### 3.3. Diuretic Utilization

**Figure 1:**
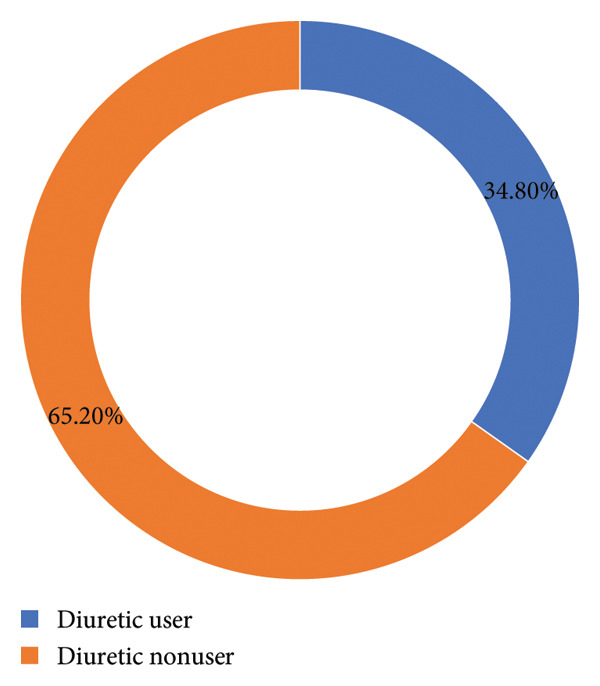
Diuretic use among the participants.

Among the studied hemodialysis patients, 163 participants (65.2%) were not taking any diuretic medication, while 87 participants (34.8%) were on diuretic therapy. Among those on diuretics, all were utilizing a loop diuretic, with 62 patients (71.3%) using furosemide and 25 patients (28.7%) using bumetanide. For the 87 patients on diuretics, the mean furosemide equivalent dose was 153.56 ± 122.57 mg per day, with doses ranging between 20 mg and 500 mg. The distribution of furosemide equivalent doses showed that 51.7% of the patients were on less than 80 mg per day, while 28.7% were on daily doses of 250 mg or more. Only 14 out of the 87 patients (16.1%) on diuretic therapy were receiving doses of at least 320 mg per day. Figure 1 and Table 3 illustrate the proportions of utilization of diuretics and the daily furosemide equivalent dose and ranges, respectively.

**Table 3:** Furosemide equivalent dose among the participants.

Variable	M ± SD or *N* (%) (*N* = 87)
Furosemide equivalent dose	153.56 ± 122.57
Range	20 mg–500 mg
Furosemide equivalent dose range (mg)	
< 80 mg per day	45 (51.7)
80 mg–249 mg per day	17 (19.5)
≥ 250 mg per day	25 (28.7)

### 3.4. Bivariate Comparison of the Sociodemographic and Hemodialysis Characteristics, and Clinical Outcomes Between Diuretic Users and Nonusers

In a bivariate analysis examining the utilization of diuretics among the studied hemodialysis patients, a significantly lower proportion of diuretic users were nondiabetic compared to nonusers (28.6% vs. 71.4%, *p* = 0.002). Diuretic users had a significantly shorter dialysis vintage compared to diuretic nonusers (3.71 ± 3.45 years vs. 6.5 ± 5.3 years, *p* < 0.001). Additionally, a significantly lower proportion of diuretic users underwent dialysis three times per week (29.3% vs. 70.7%, *p* = 0.028). In addition, absence of urine output was significantly lower among diuretic users (2.5% vs. 97.5%, *p* < 0.001). Potassium levels were also significantly lower among diuretic users (5.26 ± 0.71 mEq/L vs. 5.48 ± 0.74 mEq/L, *p* = 0.035), while average postdialysis systolic blood pressure was significantly higher among diuretic users (117.77 ± 13.30 mmHg vs. 121.17 ± 12.42 mmHg, *p* = 0.049). Tables 4 and 5 demonstrate the results of the bivariate analysis.

**Table 4:** Bivariate associations of the sociodemographic characteristics with diuretic use versus nonuse.

Variable	Diuretic nonuser (*N* = 163)	Diuretic user (*N* = 87)	*p* value
	M ± SD or *N* (%)	M ± SD or *N* (%)	
Age (years)	58.74 ± 14	61.36 ± 13.54	0.255
Gender			
Male	81 (62.3)	49 (37.7)	0.318
Female	82 (68.3)	38 (31.7)	
Marital status			
Single	18 (66.7)	9 (33.3)	0.242
Married	111 (65.7)	58 (34.3)	
Divorced/Widowed	34 (62.9)	20 (37.1)	
Occupation			
Beirut	46 (67.6)	22 (32.4)	0.860
Mount Lebanon	20 (62.5)	12 (37.5)	
Bekaa	16 (66.7)	8 (33.4)	
South Lebanon	55 (63.2)	32 (36.7)	
North Lebanon	26 (66.7)	13 (33.3)	
Education level			
Primary or less	112 (65.5)	59 (34.5)	0.629
High school	32 (68.1)	15 (31.9)	
University/postgraduate	19 (59.4)	13 (40.6)	
Household crowding index	0.956 ± 0.49	1.09 ± 0.61	0.256
Household income			
Less than 300$	76 (67.9)	36 (32.1)	0.543
300$–500$	66 (66.0)	34 (34.0)	
More than 500$	21 (55.3)	17 (44.7)	
Occupation			
Nonworking	138 (64.8)	75 (35.2)	0.743
Working	25 (67.6)	12 (32.4)	
Smoking status			
Nonsmoker	71 (64.5)	39 (35.5)	0.862
Ex‐smoker	34 (65.4)	18 (34.6)	
Current smoker	58 (65.9)	30 (34.1)	
Caffeine status			
Yes	142 (67.6)	68 (32.4)	0.266
No	21 (52.5)	19 (47.5)	
Alcohol status			
Yes	18 (69.2)	8 (30.8)	0.287
No	145 (64.7)	79 (35.3)	
Follow a potassium‐restricted diet			
Yes	59 (67.8)	28 (32.2)	0.689
Most of time	64 (62.1)	39 (37.9)	
No	40 (66.7)	20 (33.3)	
Family history			
Chronic kidney disease	50 (72.5)	19 (27.5)	0.237
Hypertension	67 (63.8)	38 (36.2)	0.694
Diabetes mellitus	58 (65.9)	30 (34.1)	0.862
Comorbidities			
Hypertension			
Yes	120 (64.9)	65 (35.1)	0.851
No	43 (66.2)	22 (33.8)	
Diabetes			
Yes	38 (50.6)	37 (49.4)	**0.002**
No	125 (71.4)	50 (28.6)	
Heart diseases			
Yes	44 (63.8)	25 (36.2)	0.769
No	119 (65.7)	62 (34.3)	
ESRD etiology			
HTN	93 (66.4)	47 (33.6)	0.254
DM	4 (36.4)	7 (63.6)	
HTN and DM	21 (55.3)	17 (44.7)	
Others	45 (73.8)	16 (26.2)	

*Note:* Bold values indicate significant values

**Table 5:** Bivariate associations of the hemodialysis characteristics and clinical outcomes with diuretic use versus nonuse.

Variable	Diuretic nonuser (*N* = 163)	Diuretic user (*N* = 87)	*p* value
	M ± SD or *N* (%)	M ± SD or *N* (%)	
Dialysis vintage (years)	6.5 ± 5.3	3.71 ± 3.45	**< 0.001**
Number of dialysis/weeks			
2x per week	59 (57.3)	44 (42.7)	**0.028**
3x per week	104 (70.7)	43 (29.3)	
Urine output			
Yes	86 (50.3)	85 (49.7)	**< 0.001**
No	77 (97.5)	2 (2.5%)	
Vascular access			
Arteriovenous fistula	144 (67.6)	69 (32.4)	0.271
Venous catheter access	19 (51.4)	18 (48.6)	
Total number of hospitalizations per patient per 6 months	0.18 ± 0.51	0.16 ± 0.45	0.794
Total number of intradialytic hypotension episodes per patient per 6 months	3.03 ± 3.14	2.74 ± 2.67	0.458
Average potassium levels (mEq/L)	5.48 ± 0.74	5.26 ± 0.71	**0.035**
Average predialysis systolic blood pressure (mmHg)	137.90 ± 18.52	138.41 ± 15.80	0.825
Average postdialysis systolic blood pressure (mmHg)	117.77 ± 13.30	121.17 ± 12.42	**0.049**
Average dry weight (kg)	69.61 ± 16.33	68.20 ± 15.12	0.505
Average interdialytic weight gain (kg)	2.40 ± 1.02	2.41 ± 0.98	0.939
Average ultrafiltration volume (L)	2.76 ± 1.00	2.75 ± 0.99	0.934

*Note:* Bold values indicate significant values

### 3.5. Predictors of Diuretics Utilization

In the multivariable logistic regression analysis, diuretic use was significantly more likely to be associated with having urine output (ORa = 23.189; 95% CI: 5.129–104.831; *p* < 0.001). Dialysis vintage was inversely associated with diuretic use (ORa = 0.892; 95% CI: 0.472–0.958; *p* = 0.046), indicating that patients with a shorter duration on dialysis were more likely to use diuretics. Additionally, higher average postdialysis systolic blood pressure was positively associated with diuretic use (ORa = 13.760; 95% CI: 10.381–18.232; *p* = 0.026) (Table 6).

**Table 6:** Multivariable logistic regression taking diuretic use as the dependent variable.

Variable	ORa	*p* value	95% CI	
			Lower bound	Upper bound
Diabetes mellitus (yes vs. no^∗^)	1.748	0.135	0.840	3.635
Number of dialysis sessions per week (3 vs. 2 times/week^∗^)	0.748	0.406	0.377	1.484
Urine output (yes vs. no^∗^)	23.189	**< 0.001**	5.129	104.831
Dialysis vintage	0.892	**0.046**	0.472	0.958
Average potassium levels	0.861	0.568	0.533	1.390
Average postdialysis systolic blood pressure (mmHg)	13.760	**0.026**	10.381	18.232

*Note:* ORa: adjusted odds ratio. Bold values indicate significant values.

^∗^Reference group.

### 3.6. Subgroup Analysis

**Figure 2:**
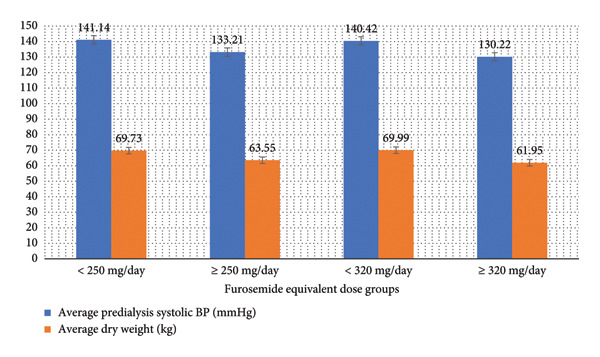
Average predialysis systolic blood pressure and dry weight by furosemide equivalent dose subgroups showing statistically significant differences (≥ 250 mg and ≥ 320 mg).

**Figure 3:**
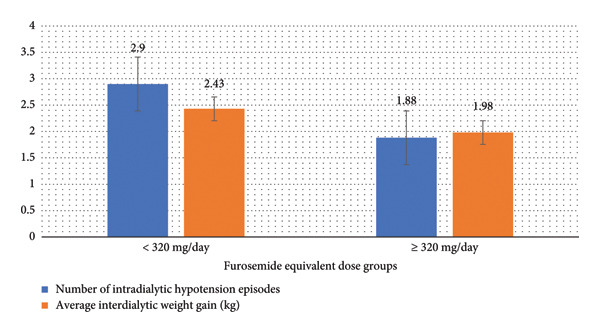
Intradialytic hypotension episodes and interdialytic weight gain by furosemide equivalent dose subgroup showing statistically significant differences (≥ 320 mg).

Further subgroup analysis among diuretic users revealed no significant difference in hemodialysis parameters and clinical outcomes between those prescribed furosemide equivalent doses below or above the cutoff point of 80 mg per day. However, with a cutoff point of 250 mg per day, diuretic users with furosemide equivalent doses equal to or greater than 250 mg per day showed significantly lower average predialysis systolic blood pressure and average dry weight (*p* < 0.05). Moreover, diuretic users with furosemide equivalent doses equal to or greater than 320 mg per day showed significantly lower intradialytic hypotension episodes, average predialysis systolic blood pressure, average dry weight, and average interdialytic weight gain (*p* < 0.05). Table 7 illustrates the bivariate analysis of furosemide equivalent dose with cutoff points of 80 mg, 250 mg, and 320 mg per day, and Figures 2 and 3 demonstrate demonstrate the statistically significant differences in predialysis systolic blood pressure, dry weight, intradialytic hypotension episodes, and interdialytic weight gain among the relevant subgroups.

**Table 7:** Bivariate analysis of furosemide equivalent dose with cutoff points of 80 mg, 250 mg, and 320 mg.

Variable	Cutoff of 80 mg per day			Cutoff of 250 mg per day			Cutoff of 320 mg per day		
	< 80 mg (*N* = 45)	≥ 80 mg (*N* = 42)	*p*‐value	< 250 mg (*N* = 62)	≥ 250 mg (*N* = 25)	*p*‐value	< 320 mg (*N* = 73)	≥ 320 mg (*N* = 14)	*p*‐value
	M ± SD or *N* (%)	M ± SD or *N* (%)		M ± SD or *N* (%)	M ± SD or *N* (%)		M ± SD or *N* (%)	M ± SD or *N* (%)	
Dialysis vintage	3.64 ± 3.68	3.76 ± 3.29	0.883	3.78 ± 3.16	3.54 ± 2.65	0.703	3.78 ± 3.66	3.50 ± 2.75	0.740
Total number of hospitalizations	0.18 ± 0.56	0.14 ± 0.35	0.620	0.17 ± 0.52	0.14 ± 0.50	0.734	0.16 ± 0.48	0.13 ± 0.35	0.771
*N* of intradialytic hypotension episodes	2.78 ± 2.62	2.70 ± 2.73	0.886	2.82 ± 2.58	2.32 ± 2.60	0.169	2.90 ± 2.22	1.88 ± 2.14	**0.039**
Average potassium levels (mEq/L)	5.18 ± 0.56	5.31 ± 0.80	0.476	5.27 ± 0.54	5.30 ± 0.59	0.706	5.23 ± 0.67	5.32 ± 0.84	0.667
Average predialysis systolic BP (mmHg)	139.20 ± 13.01	137.81 ± 17.50	0.681	141.14 ± 11.60	133.21 ± 15.20	**0.043**	140.42 ± 15.21	130.22 ± 16.01	**0.034**
Average postdialysis systolic BP (mmHg)	120.42 ± 11.60	121.70 ± 12.91	0.632	120.21 ± 12.50	121.62 ± 12.31	0.683	121.40 ± 12.25	120.21 ± 11.90	0.688
Average dry weight (kg)	69.98 ± 12.60	66.94 ± 16.75	0.369	69.73 ± 13.60	63.55 ± 16.87	**0.035**	69.99 ± 14.37	61.95 ± 16.35	**0.044**
Average interdialytic weight gain (kg)	2.51 ± 1.01	2.33 ± 0.94	0.400	2.43 ± 0.98	2.24 ± 0.95	0.712	2.43 ± 1.08	1.98 ± 0.97	**0.041**
Average ultrafiltration volume (L)	2.78 ± 1.03	2.73 ± 0.97	0.805	2.79 ± 0.94	2.74 ± 0.98	0.483	2.70 ± 0.98	2.88 ± 1.03	0.469

*Note:* Bold values indicate significant values

Abbreviation: BP = blood pressure.

## 4. Discussion

Unlike studies conducted in high‐resource settings, this analysis provides insight into real‐world diuretic use patterns in Lebanon, where dialysis protocols and drug access differ substantially. The present study demonstrated a relatively high prevalence of diuretic utilization among hemodialysis patients in Lebanon, primarily furosemide and bumetanide. Diuretic use was associated with having a residual kidney function, shorter time on dialysis, and higher postdialysis systolic blood pressure. Higher doses of diuretics of 250 mg and more per day were associated with lower predialysis blood pressure and dry weight. Furthermore, patients on 320 mg daily doses and more had fewer episodes of intradialytic hypotension episodes and lower predialysis systolic blood pressure, dry weight, and interdialytic weight gain.

Our findings revealed that around one‐third of the studied hemodialysis patients were utilizing a diuretic. A previous study reported an international overall prevalence of diuretic use of 32.6% at the time of dialysis initiation with a sharp decline in utilization to 10.7% after 2 years on dialysis therapy [16]. With an average dialysis vintage of around 3.7 years in our sample, a 34.8% diuretic utilization is considered relatively high. Diuretic use in hemodialysis is less common in some regions, where its prevalence was 13% in the United States, 21.5% in Europe, and 18.7% in Japan [16, 21]. It is believed that residual kidney function declines rapidly after starting maintenance hemodialysis, which results in early discontinuation of diuretic therapy [31, 32]. Nevertheless, it was documented that around 30% of patients still report a urine output of more than 250 mL/day after 1 year [33]. In addition, patients with residual kidney function on diuretic therapy have almost twice the odds of retaining the residual kidney function after 1 year versus patients not on diuretic therapy [16]. Therefore, diuretics can be beneficial for many hemodialysis patients with residual kidney function, even after 1 year of treatment. Furthermore, the belief that dialysis alone can manage fluid overload in hemodialysis patients, the misconception that diuretics are ineffective in advanced renal disease, and the fear of side effects like ototoxicity might explain lower diuretic use in this specific population in other countries [18].

In alignment with previous studies [16, 20, 21], the only diuretic type utilized by patients in our study was loop diuretics, with the majority being on furosemide therapy. In patients with severe chronic insufficiency, thiazides have limited efficacy [34], and potassium‐sparing diuretics have limited use due to the potential risk of hyperkalemia and the poor evidence to support a diuretic effect in advanced chronic kidney disease [35]. Hence, loop diuretics, and in particular furosemide, are the most commonly utilized among hemodialysis patients, especially because their pharmacokinetics are not modified in those patients [18, 34]. However, bumetanide should also be considered, given the possible decreased risk of ototoxicity and more reliable absorption [18].

This study revealed a mean furosemide equivalent dose of 153.56 ± 122.57 mg per day. In severe renal insufficiency, the daily oral dose of furosemide for maximal natriuresis is 320–400 mg [26]. In addition, another study demonstrated that administering a daily dose of 250–1000 mg of furosemide resulted in a 60% increase in urine volume in chronic hemodialysis patients, although this response diminished over time [30]. However, in our study, only 28.7% of diuretic users were receiving furosemide equivalent daily doses of 250 mg or more, and 16.1% were receiving daily doses of at least 320 mg. Therefore, despite a relatively high prevalence of diuretic utilization in this study, it is clear that the participants were not taking the recommended dose of furosemide to reach its therapeutic effect. Consistent with earlier findings, diuretic dosing among hemodialysis patients has been shown to vary considerably, with many prescribed lower furosemide‐equivalent doses than those advised for nondialysis chronic kidney disease populations. [21]. This could be due to concerns about the safety and tolerability of loop diuretics. Furosemide at high doses can cause ototoxicity and bullous dermatosis [36, 37]. Nevertheless, ototoxicity has not been observed with oral furosemide administered at recommended doses but rather with intravenous administration or concomitant ototoxic drugs [22, 34]. Despite this, uncertainty about optimal dosing remains.

The comparison between diuretic use and nonuse in this study revealed that diuretic users are much more likely to have a residual kidney function, as indicated by positive urine output. This finding aligns with results from previous studies [16, 21] and the available evidence that supports loop diuretic use among hemodialysis patients with residual kidney function [18, 34]. Urine output in our study was assessed subjectively by checking the medical records rather than by collecting urine for 24 h. Nonetheless, a recent study suggests that some, but not all, patients who report at least 1 cup of urine output per day may experience increased urine output in response to oral furosemide [25]. In addition, diuretic users in this study have significantly shorter dialysis vintage. This is mainly due to the decline in residual kidney function over time, which necessitates the discontinuation of diuretic therapy [31]. Furthermore, a significantly higher proportion of diuretic users had a lower number of dialysis sessions per week, slightly lower potassium levels, and higher postdialysis systolic blood pressure. These findings could be attributed to the presence of residual kidney function among diuretic users and the impact of increased urine output induced by diuretics on managing fluid overload and potassium levels. Khedr et al. reported that hyperkalemia was significantly lower in patients with residual kidney function compared to anuric patients [38]. Also, increased urine output in hemodialysis patients with residual kidney function permits better fluid status control and a lower ultrafiltration rate during the dialysis session, which eventually leads to a lower number of dialysis sessions needed and higher postdialysis systolic blood pressure [39]. Multivariable logistic regression significantly identified only 3 main predictors of diuretic utilization in hemodialysis patients, including the presence of urine output, shorter dialysis vintage, and higher postdialysis systolic blood pressure, whereas potassium level and the number of dialysis sessions per week were insignificant, indicating the presence of confounding bias.

Contrary to previous studies [16, 20], our findings revealed no significant difference in other clinical outcomes, including number of hospitalizations, number of intradialytic hypotension episodes, average predialysis systolic blood pressure, average dry weight, average interdialytic weight gain, and average ultrafiltration volume between diuretic users and nonusers. Such results are mostly due to the inadequate and insufficient dosing of furosemide in our study. Similar results were demonstrated by a recent study conducted among hemodialysis patients in the United States, where no changes in interdialytic weight gain, ultrafiltration rates, and target weight postdialysis were detected [25]. These were explained by maximum average daily furosemide doses of 69–320 mg, with almost 20% of participants receiving a maximum dose of less than 120 mg/day [25].

To explore more the impact of inadequate dosing of loop diuretics on clinical outcomes of hemodialysis patients, a subgroup analysis among diuretic users was performed. It revealed no significant differences in hemodialysis parameters and clinical outcomes when comparing those prescribed lower versus higher furosemide equivalent daily doses, except at specific cutoff points. Patients on daily doses of 250 mg or more had significantly lower average predialysis systolic blood pressure and average dry weight, indicating more effective fluid removal and blood pressure control with higher diuretic doses. Those on daily doses of 320 mg or more also had significantly lower intradialytic hypotension episodes, predialysis systolic blood pressure, dry weight, and interdialytic weight gain, suggesting that higher diuretic doses may confer additional benefits in managing fluid overload and maintaining hemodynamic stability.

### 4.1. Practical Implications

Despite the significant utilization of diuretics among hemodialysis patients, it was shown that many of those patients were not receiving optimal doses. This is mostly due to concerns about side effects and dosing uncertainties. However, it was revealed that diuretic doses higher than 250 mg per day may provide additional benefits in managing fluid overload. Therefore, clinicians should consider higher diuretic doses for hemodialysis patients with residual kidney function and shorter dialysis vintage to enhance fluid management and maintain hemodynamic stability. Additionally, standardized, evidence‐based dosing guidelines for this population are of utmost importance to guide clinicians in individualizing patient care and optimizing diuretic use.

### 4.2. Strengths and Limitations

The study exhibits several strengths and limitations. Among the strengths, this study is the first in Lebanon to provide a thorough assessment of the utilization of diuretics among hemodialysis patients. The multicenter design enhances the generalizability of the findings by including eight large dialysis centers across various regions of Lebanon. With a sample size of 250 participants, the study exceeds the minimum requirement, enhancing statistical power and reliability. Additionally, the study measures a wide range of sociodemographic, clinical, and dialysis‐related parameters, allowing for a comprehensive analysis of factors associated with diuretic use.

However, the study also has limitations. Its retrospective design may introduce recall bias and limit the ability to establish causality. Medication adherence was not assessed and could not be guaranteed, increasing the likelihood of misclassification bias. Also, urine output was evaluated subjectively rather than through lab tests, which may affect the accuracy of the data collected. A low acceptance rate from the randomly chosen hospitals might affect the representativeness of the sample. In addition, data were limited to the past 6 months, potentially missing long‐term trends and outcomes. Also, unmeasured confounding variables could still influence the observed associations. Lastly, regional differences in practices and patient demographics might introduce heterogeneity not fully accounted for in the analysis. Accordingly, further research is suggested to override these limitations.

## 5. Conclusion

This study offers valuable insights into the utilization of diuretics among hemodialysis patients in Lebanon. Despite a relatively high prevalence of loop diuretic use, many patients do not receive adequate doses to achieve optimal therapeutic effects. Diuretic users are more likely to have a residual kidney function, a shorter dialysis vintage, and a higher postdialysis systolic blood pressure compared to nonusers. However, most hemodialysis‐related parameters and clinical outcomes do not differ between diuretic users and nonusers. Higher diuretic doses, particularly daily furosemide doses of 250 mg or more, are associated with improved clinical outcomes, emphasizing the need for further research to optimize dosing practices in this population. The study’s retrospective design, potential recall and misclassification biases, and variability in dosing practices highlight the need for future prospective studies to investigate the efficacy, safety, and optimal dosing of diuretics in individuals with kidney failure who are dependent on hemodialysis.

**Nomenclature**
ANOVA: One‐way analysis of variances
BP: Blood pressure
CI: Confidence interval
ESRD: End‐stage renal disease
ORa: Adjusted odds ratio
SPSS: Statistical Package for Social Sciences

## Ethics Statement

This study was conducted in accordance with the ethical standards of the Declaration of Helsinki (2013 revision). Ethical approval was obtained from the Ethics and Research Committee of the School of Pharmacy at the Lebanese International University (Approval No. 2023ERC‐130‐LIUSOP, January 2, 2023) and from the institutional review boards of all participating hospitals. Written informed consent was obtained from all participants prior to inclusion. Patient confidentiality and data privacy were strictly maintained throughout the study.

## Consent

Please see Ethics Statement.

## Disclosure

All authors approved the final manuscript.

## Conflicts of Interest

The authors declare no conflicts of interest.

## Author Contributions

I.F., M.D., J.S., F.S., and M.R. conceptualized the study. I.F. had the responsibility of the project administration, planned the methodology, and carried out the statistical analyses. F.E. supervised data collection. I.F. and F.E. wrote the manuscript text. All authors reviewed and edited the text.

## Funding

The authors received no specific funding for this work.

## Data Availability

The datasets used and/or analyzed during the current study are available from the corresponding author on reasonable request.
